# Adenine Phosphoribosyltransferase Deficiency: A Rare Cause of Recurrent Urolithiasis

**DOI:** 10.1089/cren.2017.0015

**Published:** 2017-04-01

**Authors:** Ata Jaffer, Adrian Joyce, Philip Koenig, Chandra Shekhar Biyani

**Affiliations:** ^1^ Department of Urology, St James's University Hospital, Leeds Teaching Hospital NHS Trust, Leeds, United Kingdom.; ^2^ Department of Urology, Airedale General Hospital, Airedale NHS Foundation Trust, Keighley, United Kingdom.

**Keywords:** adenine phosphoribosyl transferase deficiency, urolithiasis, 2,8-dihydroxyadenine

## Abstract

***Background:*** Recurrent urolithiasis is troublesome for both patient and clinician, and in most cases, an underlying cause is not found. An important and underdiagnosed cause is adenine phosphoribosyltransferase (APRT) deficiency that gives rise to 2,8-dihydroxyadenine (2,8-DHA) stones. If diagnosed early, patient morbidity as well as the financial cost of treating stone recurrence can be avoided with simple medical therapy.

***Case Presentation:*** A 36-year-old white, Caucasian male with recurrent urolithiasis was found to have 2,8-DHA stones. This was difficult to manage, as these stones were often large, bilateral, matrix in structure, and translucent on plain X-rays. He underwent a multitude of interventions including both retrograde and anterograde endoscopic approaches as well as extracorporeal shock wave lithotripsy. The specific stone type was eventually discovered through infrared spectroscopy and he was promptly commenced on allopurinol, which significantly improved his stone burden and frequency of presentation with renal colic.

***Conclusion:*** APRT deficiency is underdiagnosed given the estimated prevalence of 1/50,000–1/100,000, however, with less than 300 reported cases worldwide. This is likely because of both a lack of awareness of the disorder among clinicians and the challenges of identifying 2,8-DHA stones. Increasing awareness of 2,8-DHA urolithiasis among urologists as well as physicians is, therefore, key in tackling this condition.

## Introduction and Background

Recurrent urolithiasis is a condition that causes a significant level of morbidity for patients and frustration for urologists. A few well-documented, rare causes have been identified, but most cases remain without a known underlying cause. Adenine phosphoribosyltransferase (APRT) deficiency is a rare inborn error of metabolism first described in the United Kingdom in 1976.^1^ It is inherited as an autosomal recessive trait and the gene is located on chromosome 16. APRT is a salvage enzyme that normally catalyzes the conversion of adenine to adenine monophosphate. Deficiency results in adenine accumulation with excretion of 2,8-dihydroxyadenine (2,8-DHA) in the urine. 2,8-DHA is extremely insoluble in urine at any pH,^2^ and as a result gives rise to crystalluria that presents either as urolithiasis or chronic kidney failure. Estimated prevalence of APRT deficiency is thought to be between 1/50,000 and 1/100,000 with less than 300 published case reports,^3^ it is likely that the condition is underdiagnosed. We report a case of a patient who was found to have 2,8-DHA stones after repeat admissions with urolithiasis.

## Presentation of Case

**FIG. 1.**
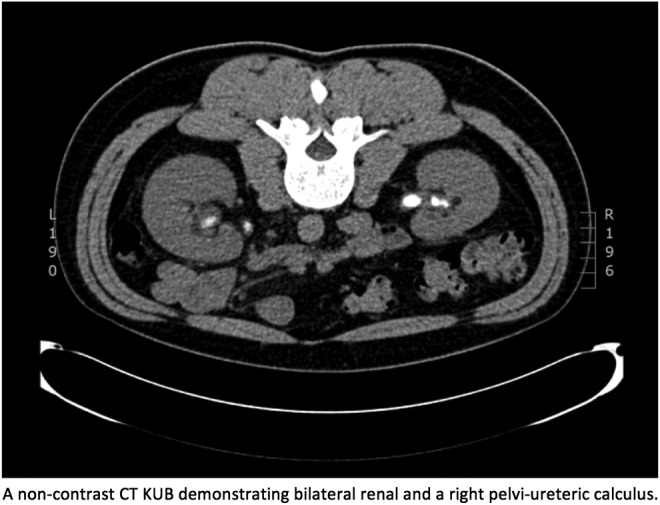
A noncontrast CT KUB showing bilateral renal and a right pelviureteral calculus. KUB, kidney, ureter, and bladder radiograph.

A 36-year-old white, Caucasian male presented in July 2008 with right-sided loin to groin pain. He had a soft abdomen with right flank tenderness. His blood results were unremarkable; however, a urine dipstick was positive for blood and leucocytes. He, therefore, underwent work-up for urolithiasis with a CT kidney, ureter, and bladder radiograph revealing a 7 mm right distal ureteral calculi with further right renal calculi. A ureterorenoscopy was initially attempted; however, this was difficult as the patient had a tight bulbourethral stricture. His stones were, therefore, managed with percutaneous nephrolithotomy. Over the next 2 years, he presented to the Urology Department several times with episodes of urolithiasis, which were difficult to manage as they were often bilateral, large, and radiolucent on plain X-rays and were matrix-like in character (Fig. 1). After an in-depth stone analysis in 2010 that included infrared spectroscopy, 2,8-DHA stones were diagnosed. He was promptly commenced on allopurinol, which reduced the frequency of recurrences and overall stone burden.

## Discussion and Conclusion

**FIG. 2.**
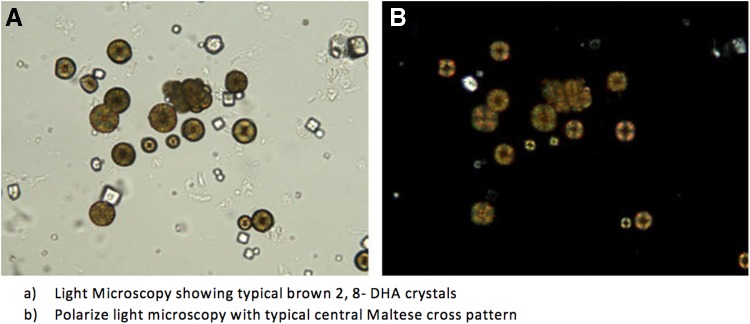
**(A)** Light microscopy showing typical brown 2,8-DHA crystals. **(B)** Polarized light microscopy with typical central Maltese cross pattern. With permission from V. Edvardsson, MD, The APRT Deficiency Program of the Rare Kidney Stone Consortium (www.rarekidneystones.org/dha/), Landspitali—The National University Hospital of Iceland, Reykjavik, Iceland. APRT, adenine phosphoribosyltransferase; DHA, dihydroxyadenine.

APRT deficiency is a rare inborn error of metabolism, and although less than 300 case reports have been reported, the prevalence is likely to be much higher. A lack of knowledge of this disorder among clinicians and urologists alike is due, in part, responsible for the small number of identified cases. Misdiagnosis for uric acid stone is also another contributing factor. It is imperative that advanced stone analysis methods such as infrared or ultraviolet spectroscopy at pH 2–10 should be used, as basic stone analysis methods will not differentiate 2,8-DHA stones from uric acid stones. On polarized light microscopy, 2,8-DHA crystals appear round and reddish brown, with a characteristic central Maltese cross pattern (Fig. 2). Almost all 2,8-DHA stones are caused by APRT deficiency and this can be formally diagnosed by assaying APRT activity in red cell lysate or through genetic testing in populations in which the deficiency has been highlighted.^4^ If diagnosed early, the condition is treatable. Edvardsson et al. observed that allopurinol dosage of 5–10 mg/kg·day is effective in preventing most recurrences of stones. 2,8-DHA solubility is not altered by pH and, therefore, alkalization is not recommended. A low purine diet, however, as is recommended for uric acid stones, has been shown to be beneficial. In conclusion, a high index of suspicion of APRT deficiency should be held for young patients presenting with recurrent urolithiasis, as early management is critical in reducing the morbidity associated with this condition as well as the financial burden on the health service.

## Abbreviations Used

2,8-DHA: 2,8-dihydroxyadenine
APRT: adenine phosphoribosyltransferase
CT: computed tomography
KUB: kidney, ureter, and bladder radiograph
